# A practical guide for the generation of model-based virtual clinical trials

**DOI:** 10.3389/fsysb.2023.1174647

**Published:** 2023-06-16

**Authors:** Morgan Craig, Jana L. Gevertz, Irina Kareva, Kathleen P. Wilkie

**Affiliations:** ^1^ Department of Mathematics and Statistics, Université de Montréal, Montréal, QC, Canada; ^2^ Sainte-Justine University Hospital Research Centre, Montréal, QC, Canada; ^3^ Department of Mathematics and Statistics, The College of New Jersey, Ewing, NJ, United States; ^4^ EMD Serono Inc., Merck Group, Billerica, MA, United States; ^5^ Department of Mathematics, Toronto Metropolitan University, Toronto, ON, Canada

**Keywords:** virtual patients, virtual clinical trials, parameter estimation, model identifiability, sensitivity analysis, mathematical modelling

## Abstract

Mathematical modeling has made significant contributions to drug design, development, and optimization. Virtual clinical trials that integrate mathematical models to explore patient heterogeneity and its impact on a variety of therapeutic questions have recently risen in popularity. Here, we outline best practices for creating virtual patients from mathematical models to ultimately implement and execute a virtual clinical trial. In this practical guide, we discuss and provide examples of model design, parameter estimation, parameter sensitivity, model identifiability, and virtual patient cohort creation. Our goal is to help researchers adopt these approaches to further the use of virtual population-based analysis and virtual clinical trials.

## 1 Introduction

Virtual populations (VPs) and *in silico* clinical trials (also called virtual clinical trials) are relatively new mathematical modeling techniques that are being increasingly used in the fields of quantitative systems pharmacology and mathematical oncology (Scott, 2012; Kim et al., 2016; Polasek and Rostami-Hodjegan, 2020; Wang et al., 2020; Jenner et al., 2021b; Zahid et al., 2021; Cardinal et al., 2022). Traditionally, modeling and simulation have focused on understanding biological processes and systems, sometimes with the aim of supporting drug development (Holford et al., 2000; Holford et al., 2010; Scott, 2012; Kim et al., 2016; Polasek and Rostami-Hodjegan, 2020; Wang et al., 2020; Jenner et al., 2021b; Zahid et al., 2021; Cardinal et al., 2022). *In silico* clinical trials extend the tools and scope of modeling and simulation with the goal of predicting the heterogeneous effects of drugs on populations of individuals. In certain disciplines, virtual clinical trials can make use of statistical inference instead of an underlying mathematical model (Van Camp et al., 2023); in this guide we focus solely on *in silico* trials based on mathematical and computational modelling.

Specific applications of *in silico* clinical trials include refining dose projections for new drugs before they enter the clinic, studying inter-patient variability in treatment response, stratifying patient populations to identify treatment responders *versus* non-responders, and assessing potential drug combinations or alternate treatment regimes. Thus, the questions virtual trials are primed to address closely align with the dose optimization and selection goals set forth by the U.S. Food and Drug Administration’s Project Optimus initiative (FDA, 2022) (USFDA, 2023). VP-based analysis can also be viewed as a bridge between the ubiquitous standard-of-care approach designed around the “average patient” and fully personalized therapy. Currently, most clinical trials are designed around the average patient, which can result in toxicity or lack-of-efficacy for certain patients. Exceptions are starting to emerge, including the I-SPY trials that utilize patient imaging and tumor profiles to adaptively select the most promising investigational drug to pair with standard-of-care treatments for each patient in the trial (Perlmutter, 2011).

Despite recent progress, many challenges remain in realizing the promises of personalized therapy. From a modeling and simulation perspective, some of the challenges of personalization are highlighted in a recent study by Luo et al. (Luo et al., 2022). In this work, a mathematical model of murine cancer immunotherapy that was previously validated against the average of an experimental dataset was employed to make personalized treatment predictions for genetically identical animals. The study found that one could not confidently use the model for personalized treatment design due to parameter identifiability issues that emerged when shifting the model from the average context to the individual context. Given the current scientific, computational, and financial challenges of personalized therapy, tools that provide a better understanding of population variability and its impact on treatment response are needed. For this reason, virtual population analysis is becoming an indispensable tool for advancing our understanding of treatment response at both the individual and population level.

There are various methods for defining virtual populations and for running *in silico* clinical trials. Different approaches have been taken by different authors and been applied to a variety of questions, ranging from the standard population pharmacokinetics analysis used in drug discovery and development (Mould and Upton, 2012; Mould and Upton, 2013), to simulating treatment impact over a range of patient-specific characteristics. Here we will generally focus on the latter approach via mechanistically based VP generation.

Independent of the approach used to define a VP, one can view VP-based analysis as a form of sensitivity analysis wherein we select characteristics of a population that we believe might affect responses, introduce variability in one or more of the associated parameters (i.e., drug clearance or impact on a biomarker), and use a mathematical model to predict variability in outcomes. Typically, we assume that selected characteristics deviate from the average to some degree, ensuring that VPs represent a heterogeneous population. However, there is no consensus in the field regarding how one designs an appropriate model, identifies VPs characteristics, and introduces variability in those characteristics, so that a virtual clinical trial is best-positioned to answer the motivating treatment-related question.

In this paper, we introduce a step-by-step best practice guide for researchers looking to extend their modeling and simulation work by designing virtual patient cohorts for *in silico* clinical trials. This methodological guide, developed to be broadly applicable to a wide range of questions and software platforms, complements previous overviews of virtual clinical trials in the literature, see for instance (Pappalardo et al., 2019; Alfonso et al., 2020; Cheng et al., 2022). We begin by considering how to build a fit-for-purpose model to address the aim of a virtual clinical trial before exploring a range of techniques available to parametrize such models using available biological, physiological, and treatment-response data. We next explain the important role that sensitivity analysis (quantifying changes in model output as a function of model input) and identifiability analysis (quantifying what we can and cannot say about model parameters given available data) play in selecting the characteristics of your virtual patients. This leads us to a discussion of various iterative processes that have been introduced for designing a virtual population so that you can conduct your *in silico* clinical trial. We conclude with a discussion of the benefits and limitations of these computational methods as a complement to clinical trials.

## 2 Steps for designing virtual patient cohorts for in silico clinical trials

**FIGURE 1:**
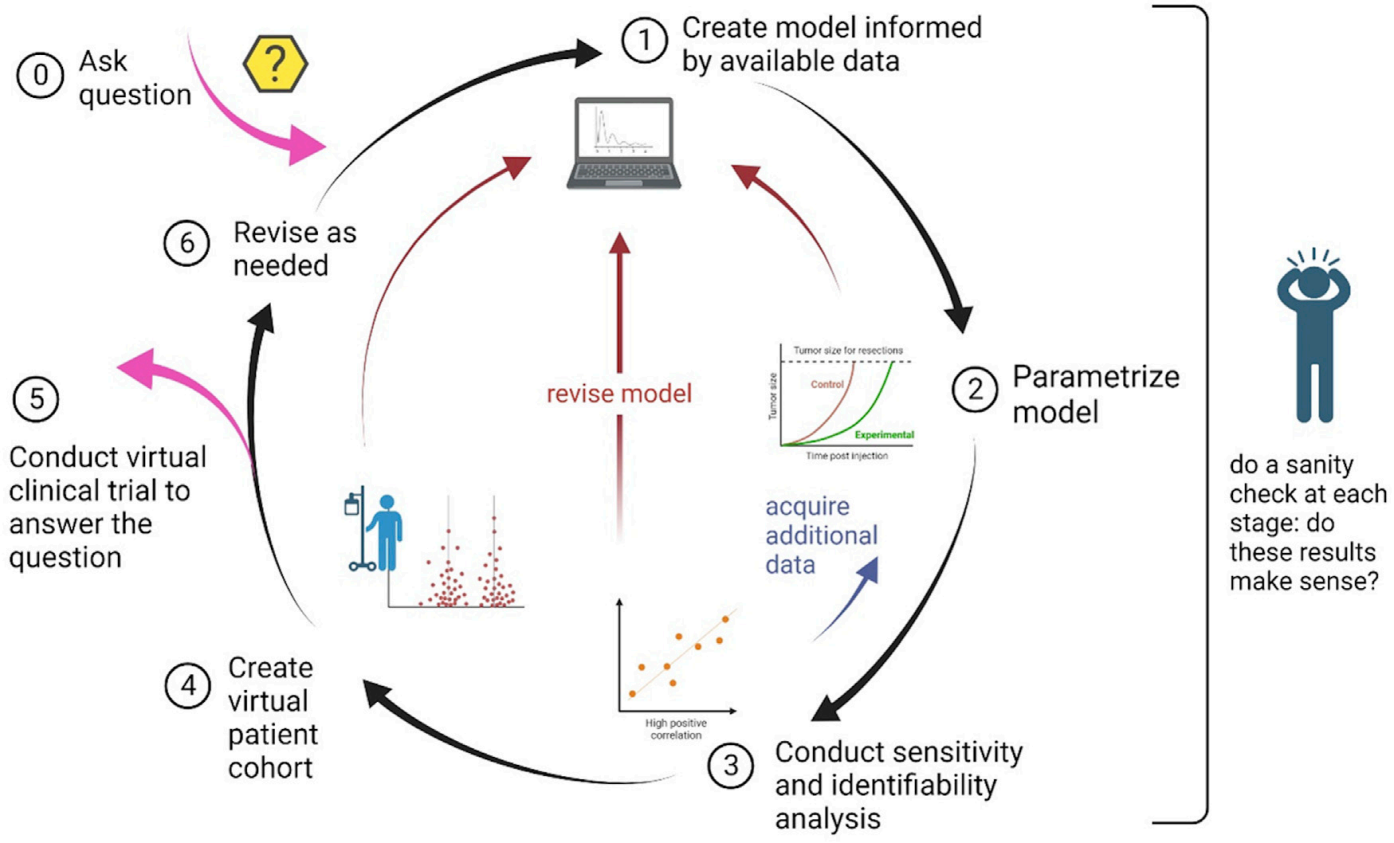
Schematic of the process to design and implement an *in silico* clinical trial. The nature of the process is cyclical, with the possibility of steps being revisited as needed.

In this section we detail the step-by-step guidelines for conducting an *in silico* clinical trial. A schematic summarizing these steps, and highlighting the iterative nature of the process, is found in Figure 1.

### 2.1 Step 1: Building a model for VP analysis

Model-based analysis has become an integral part of drug discovery, development, and optimization. A major challenge in model-based analysis is designing a model with not only the correct structure, but also the appropriate level of mathematical detail. Mechanistic mathematical models can incorporate an immense amount of detail by, e.g., including elaborate descriptions of drug pharmacokinetics or signal transduction pathways important for tumor growth and response to the treatment under study. Such complex models may allow for a better analysis of the underlying biological mechanisms, and thus may be well-suited to understand what causes patients to either respond, or not respond, to the treatment. However, beyond computational demands, higher levels of detail come with costs. For example, there is high potential for error propagation in predictions if sufficient data are not available for model parametrization.

On the other end of the spectrum are phenomenological models. These models are generally not concerned with detailed mechanisms and are instead focused on capturing the general behavior of a system using a small number of variables and parameters. Such models are much easier to parametrize and analyze, though they cannot provide the detailed mechanistic insights of larger and more complex models. Bridging these two extremes are intermediate-sized fit-for-purpose models that incorporate mechanistic details for the most important subcomponents of the system. In such models, the components deemed to be less important for the drug of interest, or for which adequate information is available, are represented using more phenomenological equations. Therefore, the choice of model structure and level of mathematical detail must be tailored to the aims of your specific *in silico* clinical trial. Model selection techniques, including information criteria (Dziak et al., 2020) and testing model predictions on held out data (Brady and Enderling, 2019), can also be valuable tools when trying to identify the most parsimonious model to describe experimental data from a set of plausible models (Liu et al., 2021; Cárdenas et al., 2022).

The aspects of model development one must consider can be generally divided into pharmacokinetic (PK) and pharmacodynamic (PD) components. Drug pharmacokinetics refer to the change of drug concentration over time (i.e., what the body does to the drug) and can typically be captured using two main classes of parameters that describe the volume of distribution and the clearance rate (Mould and Upton, 2013). For drugs that are administered orally or subcutaneously, it is also necessary to estimate the rate of drug absorption. PKs are typically described using compartment-based models that capture the movement of the drug from its initial (typically plasma) compartment into other regions of the body before eventually being cleared by the system. An important consideration for building an accurate PK model is determining how many compartments (mathematical equations) are needed to adequately describe available preclinical data. It is of note that while PK parameters are typically determined from preclinical experiments, a number of well-developed methods exist to scale PK parameters to humans (Mager et al., 2009; Huang and Riviere, 2014; Davies et al., 2020).

Compared to PK modeling, quantifying the pharmacodynamic effects of a drug is more challenging. PD models share the goal of predicting treatment safety and/or efficacy (Mould and Upton, 2013). As an example, in preclinical oncology efficacy is typically benchmarked by tumor growth inhibition (TGI) curves obtained from mouse models. It is often at this stage where an experienced modeler, in combination with the experimental collaborators, needs to make choices about how much complexity to build into their model. TGI curves provide little data to parametrize complicated models, so the availability of other experimental measurements should guide how much mechanistic detail to include, and for which model components. It is important to note that as challenging as it is to build the right-sized PD model using TGI data in mice, the challenges are much greater when working with human data. One cannot predict human dynamics based on mouse TGI curves, and as TGI curves are rarely available for humans, one must rely on biomarkers that can be readily collected to build and validate PD models for humans.

### 2.2 Step 2: Model parametrization

After constructing a mathematical model to answer the motivating question, the next step is to parametrize the model. For a given model M with r=m+n parameters, m parameters will be fixed at values from the literature (which can be represented in the vector $q=\left(q_{1},\ldots ,q_{m}\right)$ ), and n parameters will vary per virtual patient (which can be represented in the vector $p=\left(p_{1},\ldots ,p_{n}\right)$ ). Then, the *i*th virtual patient is represented by the variable vector $p_{i}$ , and their model-predicted response is found by solving $M\left(q,p_{i},t\right)$ . While it may be tempting to assume that all model parameters vary and have an underlying distribution of values, such an approach may skew results of the analysis by introducing bias or assigning weight to characteristics that are not relevant to the question of interest.

While selecting which parameters to fix and which to form the basis of the VP-based analysis can be challenging, there are several quantifiable metrics that can be applied to help make this decision. Parameters that are fixed for the whole population (i.e., q ) might include:

• parameter values for which there is strong and consistent experimental/literature support, for instance drug half-life,
• parameters that have low sensitivity to perturbation, or
• parameters that are a part of the model but not necessarily essential to the specific aims of the virtual trial and motivating question.

In contrast, parameters that may capture population variability (i.e., p ) might include:

• parameters that have high sensitivity to perturbation, a common example being the intrinsic tumor growth rate,
• parameters that capture biological aspects demonstrated by the available data, and thus can be estimated by fitting to the data, or
• parameters that describe biological features that are known to be highly heterogeneous in a population.

In practice, model parameters chosen to represent the virtual patient cohort for an *in silico* clinical trial ( p ) *versus* those that are kept constant ( q ) might change as one begins the parametrization process and learns about sensitivity and identifiability (explored further in Step 3). The iterative nature of determining which parameters to fix and which to fit is highlighted in the schematic shown in Figure 1.

Model parameters can be fit in one of two ways: either simultaneously or hierarchically. If most model parameters can be fixed, then, depending on the available data, it may be possible that good estimates can be found by fitting the entirety of p simultaneously. However, if p contains a large number of parameters, and the available data is appropriately decoupled, then a hierarchical data-fitting approach may be preferred (Wares et al., 2015; Jenner et al., 2021a; 2021b; Cho et al., 2023). For example, assume that model M describes tumor response to treatment, where parameter $p_{1}$ describes tumor growth rate, and parameter $p_{2}$ describes tumor sensitivity to treatment. In this case, the best way to estimate model parameters would be to first fit the model to the control (untreated tumor) data to estimate parameter $p_{1}$ . This value is held fixed in the *i*th VP parameter vector $p_{i}$ , and the model is then fit to the treated data to estimate $p_{2}$ . This hierarchical approach will improve identifiability for both parameters as compared to simultaneously fitting both parameters to only the treated dataset (Cho et al., 2023). However, while it is technically possible to pool data from multiple experiments, it is preferrable to ensure that both the control and the treatment curves were collected under the same experimental conditions. Sometimes, only one dataset is available, as, for example, in a trial where having a placebo arm would be unethical. In this case, it is best to try reducing model parameter redundancy before attempting to estimate their values from data.

Once the patient-specific model parameters p have been selected, and the choice of simultaneous or hierarchical fitting is made, an optimization method for identifying the best-fit parameters must be chosen. Many methods are available for this task, each with their own benefits and limitations. Local optimization algorithms are generally less computationally expensive than global algorithms, though local algorithms are more likely to converge to a suboptimal solution (Shoarinezhad et al., 2020). In general, parameter estimation is an ill-posed inverse problem where, given dataset D and mathematical model M , we seek to find the parameter vector $p_{i}$ such that the model’s prediction $M\left(q,p_{i},t\right)$ best fits the data with respect to some measure. This problem is unstable with respect to noise in the data, and real-world measurements are inevitably noisy.

Perhaps the simplest method of curve fitting is the method of least squares. In MATLAB, *lsqnonlin* minimizes the sum of squared residuals by using approximate representations of the gradient and the Hessian matrix (Mathworks, 2023b). This is implemented in a wrapper *lsqcurvefit* to perform data fitting using a nonlinear model (Mathworks, 2023a). Implementation in R can be achieved using the function *nls*. While in practice these methods typically give acceptable results, one should be aware that the least squares approach assumes that the residuals are normally distributed. Further, a major limitation of least squares is that outliers can have a significant impact on the resulting fit. Additionally, the optimization is local to the initial parameter guess $p^{0}$ . For this reason, it is necessary to start with a reasonable initial parameter guess to achieve the globally best parameter set, which is something that can be difficult in practice.

To circumvent the challenges of the dependency on the initial parameter guess, one can perform many repetitions of the fitting process with randomized initial guesses to find the globally best parameter set. In this case, the initial guess $p^{0}$ could be randomly sampled from uniform distributions over biologically reasonable ranges; alternatively, the parameter space could be subdivided and $p^{0}$ can be iterated over all subdivision grid points of the parameter space. These multi-start sampling methods extend local searches to global ones. For instance, Latin Hypercube sampling, a type of Monte Carlo sampling, performs global searches by randomly sampling a parameter’s value from all subdivisions in its entire parameter space (McKay et al., 2000). It then randomly combines the samples for all parameter values together to form an ensemble of random parameter sets. Lastly, the ensemble of parameter sets is tested, and over the entire ensemble, the best fitting parameter set can be found. Continuing increases in computational power have made multi-start methods more feasible than they were in the past.

There are many other optimization methods that can be used for parameter estimation. Some examples of global methods include genetic algorithms that modify a population of potential parameter sets and at each step evolve new potential parameter sets based on the ‘parent’ sets, and Markov Chain Monte Carlo (MCMC) and simulated annealing methods, which similarly generate new potential parameter sets through random perturbation of the previous set. These types of optimization methods rely on random number generation to find the next potential candidate parameter value instead of deterministic calculations, such as those based on gradients and Hessians. Because of this randomization, these optimization methods often accept worse-fitting parameter modifications to guarantee that they eventually converge to the global best-fitting parameters.

Several optimization methods have the added feature that they identify the posterior population-level distribution of each fitted parameter, rather than only identifying a single best-fit value, which can provide valuable insight into the population-level heterogeneity of your model parameters. For instance, nonlinear mixed effects modeling is a statistical framework for identifying fixed effects (parameters that can generalize across an entire population) and random effects (parameters that differ between individuals) that are randomly sampled from a population) (Olofsen et al., 2004). Nonlinear mixed effects modeling is a parametric method, where the user must specify the structure of the posterior distribution for each fit parameter—in biology, parameters are usually assumed to be lognormally distributed to enforce non-negativity. There also exist non-parametric methods for generating posterior distributions for the fit parameters, including MCMC and the relatively new Approximate Bayesian Computation (ABC) method (Csilléry et al., 2010). In a rejective sampling ABC method, parameter $p_{1}$ is sampled from a uniform prior, and the value is either accepted or rejected by comparing the model output to data. If the value is accepted, then it is added to the set of accepted values, building up an estimate of the parameter’s posterior distribution. This probability distribution for the parameter value can then be used to inform construction of a virtual population.

With so many methods to choose from, it can be challenging to identify the “best” approach for your problem. Yet, as shown in (Luo et al., 2022), the choice of optimization method can substantially impact the conclusions of your modeling study. As a rule of thumb, as the dimensionality (i.e., number of parameters) of the fitting problem increases, the cost function that is optimized in parameter estimation increases in complexity and is more likely to have a number of local minima. Such cost functions with a bumpy landscape are better suited to genetic-type random algorithms rather than gradient-based deterministic methods, to avoid the trap of local minima (Mendes, 2001). Additionally, when fitting several parameters from the patient-specific vector $p_{i}$ , caution must be taken to not interchange these values later on. That is, optimal parameter values that are fit together should always stay together since coupled parameters cannot be independently sampled.

Once a fitting algorithm is chosen, one must also consider the form of the cost function to be optimized. Is the goal to fit to the average of the data or to fit each of the individual trajectories separately? In fact, these approaches may be combined, if one attempts to balance both objectives by fitting to an individual and the mean at the same time, say through a linear combination of error metrics (Wilkie and Vrscay, 2005):

$$cost\left(D,M,q,p_{i},\alpha \right)=\alpha {SSE}_{indiv}\left(D_{i},M,q,p_{i}\right)+\left(1-\alpha \right){SSE}_{ave}\left(D,M,q,p_{i}\right)$$ (1)

where 0≤α≤1 controls the weight assigned to the individual data trajectory. If α=1 , the optimization will fit to the individual, and if α=0 the optimization will fit to the average. Standard metrics for cost functions include the sum of squared errors (SSE), mean squared errors (MSE), root mean squared errors (RMSE), and others. When fitting to the average, costs like the SSE can be normalized by the variance at each time point. Such a normalization reduces the cost associated with a data point that has a large variance (that is, a data point we do not have much confidence in). The choice of cost function should be based on the available data and the optimization procedure.

Finally, one must consider if there are any constraints to impose on the fitted parameters. In most biological models, parameters must be constrained to be non-negative. But often it is necessary to impose a stricter lower bound on parameters (so an important biological feature is not removed from the model by setting its parameter equal to zero, for example,), or to impose an upper bound on the value of a parameter (to ensure the value is biologically plausible). Note, however, that fitted parameters generally should not take on the values of their upper or lower bounds. If this is the case, the constraints need to be adjusted, or the model revised. Another consideration is that, in some instances, relationships need to be imposed between parameters. For instance, if we have a model with a drug-sensitive and drug-resistant population, the drug efficacy for the sensitive population must be strictly greater than the efficacy for the resistant population. All told, there is an immense number of choices one makes when parametrizing a model: which parameters to fit, whether to fit them simultaneously or hierarchically, what optimization algorithm to use, what cost function to minimize, what initial guess to make for the parameters, what bounds to impose, and so on. Because of this, it is important to remember that the model parametrization step of any project will always take longer than anticipated. As a rule of thumb, estimate how long you think the parametrization step will take and then double it. Reliable model simulation results are based on solid model formulation, data collection, and parameter estimation, so this step should not be rushed!

### 2.3 Step 3: Understanding your model and parameter landscape

Once your model is parametrized, understanding the parameter landscape and model output space through sensitivity and identifiability analyses is another essential step for developing a fit-for-purpose model for your virtual clinical trial. Sensitivity analysis is used to quantify how changes in model input (parameters) affect model outputs (i.e., tumor size). This in turn informs model structure, as highlighted in the schematic in Figure 1, and may even lead to recommendations for future experimental design (Zhang et al., 2015). Sensitivity analyses can be conducted locally or globally (Qian and Mahdi, 2020).

A local sensitivity analysis focuses on a single parameter at a time. Typically, one perturbs the parameter value a relatively small amount and quantifies the impact this perturbation has on model output (Zhang et al., 2015). Suppose the best-fit parameter set $\bar{p}=\left({\bar{p}}_{1},{\bar{p}}_{2},...,{\bar{p}}_{i},...,{\bar{p}}_{n}\right)$ is known, and $Y\left(\bar{p}\right)$ is the model output at a selected time point corresponding to the best-fit parameter set (for instance, tumor volume at some terminal time point). A local sensitivity analysis of parameter ${\bar{p}}_{i}$ would perturb this parameter a small amount $\Delta p_{i}$ , giving a new parameter set $p_{i}^{*}=\left({\bar{p}}_{1},{\bar{p}}_{2},\ldots ,{\bar{p}}_{i}+\Delta p_{i},\ldots ,{\bar{p}}_{n}\right)$ . The first-order local sensitivity index $S_{i}$ for $p_{i}$ is then defined as the partial derivative of the output with respect to the input parameter $p_{i}$ (Zi, 2011). A number of methods exist to approximate this partial derivative (Zi, 2011), though the simplest uses a finite difference approximation:

$$S_{i}=\frac{\partial Y\left(p\right)}{\partial p_{i}}\approx \frac{Y\left(p_{i}^{*}\right)-Y\left(\bar{p}\right)}{\Delta p_{i}}$$ (2)

The larger the value of $S_{i}$ , the more the parameter perturbation affects model output, and thus the more locally sensitive the parameter is considered. Historically, sensitivity analyses were conducted after the system reached a steady state; however, now it is common practice to choose a meaningful timepoint, such as immediately following a treatment administration to assess parameter sensitivity. One can extend this analysis to a dynamic sensitivity, by computing $S_{i}\left(t\right)$ for $0\leq t\leq t_{final}$ , to explore the robustness of parameters over a timeframe of interest (Farhang-Sardroodi et al., 2022).

The advantage of local parameter sensitivity analysis is computational simplicity: one must only solve the model at n+1 parameter sets to quantify local sensitivity, where n is the number of parameters. More sophisticated methods are available to compute the local sensitivity index, though they require more computations (Zi, 2011). Nonetheless, local sensitivity analysis has several shortcomings. First, it can only be used when model output is approximately linear within the specific region of interest. Further, it only quantifies the impact of changing one parameter at a time and therefore cannot evaluate the impact of parameter interactions (Zhang et al., 2015). Finally, it does not allow for the determination of whether there is more than one region of parameter space that near equally-well describes the data, as the analysis is performed locally about a particular point (the best-fit parameters, if known) in parameter space.

As virtual clinical trials must be composed of a heterogeneous group of virtual patients, it is likely too restrictive to only quantify sensitivity local to the best-fit parameter set. A more holistic view of the parameter space can be attained through a global sensitivity analysis. A range of approaches exist to perform such analyses, and the reviews in (Zi, 2011; Qian and Mahdi, 2020) summarize a number of these methods and their associated computational costs. All these methods overcome the shortcomings of local sensitivity analysis by quantifying the sensitivity of model output with respect to large variations in the input parameters. Here we will focus on a computationally intensive but powerful class of global sensitivity methods: variance decomposition techniques.

Variance decomposition techniques, including Sobol sensitivity analysis and the Fourier amplitude sensitivity test (FAST), quantify the contributions of a model parameter (or a combination of parameters) to the output variance. We will focus on Sobol’s method, while noting there are excellent resources to learn about FAST and extended FAST (eFAST) (Marino et al., 2008). To understand the Sobol sensitivity analysis method, consider the set of input parameters $p=\left(p_{1},p_{2},...,p_{i},...,p_{n}\right)$ and view each $p_{i}$ , after rescaling into the range [0,1], as a mutually independent uniformly distributed random variable. Interpreting the parameters in this way means the model output $Y\left(p\right)$ is a random variable with mean $Y_{0}$ and variance D , where

$$D=\int Y{\left(p\right)}^{2}dp-Y_{0}^{2}=\int Y{\left(p\right)}^{2}dp-{\left(\int Y\left(p\right)dp\right)}^{2}$$ (3)

And each multiple integral is evaluated over the rescaled domain on each dimension (Zhang et al., 2015).

As the name implies, variance decomposition methods decompose variance of the output $Y\left(p\right)$ into the contribution from single parameters, pairwise combinations of parameters, and so on:

$$Y\left(p\right)=Y_{0}=\sum_{i=1}^{n}Y_{i}\left(p_{i}\right)+\underset{i<j}{\sum }Y_{ij}\left(p_{i},p_{j}\right)+...+Y_{1...n}\left(p_{1},...,p_{n}\right),$$ (4)

where model output $Y_{i}$ is a function of $p_{i}$ , $Y_{ij}$ is a function of $p_{i}$ and $p_{j}$ , etc. This can be shown (see (Zhang et al., 2015)) to be equivalent to decomposing the total variance D as

$$D=Y_{0}=\sum_{i=1}^{n}D_{i}+\underset{i<j}{\sum }D_{ij}+\ldots +D_{1\ldots n},$$ (5)

where $D_{i_{1}\ldots i_{k}}=\int Y_{i_{1}\ldots i_{k}}^{2}dp_{i_{1}}\ldots {dp}_{i_{k}}$ is called the partial variance of Y corresponding to the subset of parameters $\left.{\left\{i\right.}_{1},\ldots ,i_{k}\right\}$ . With these partial variances, the corresponding sensitivity indices can be defined as

$$S_{i_{1}\ldots i_{k}}=\frac{D_{i_{1}\ldots i_{k}}}{D}.$$ (6)

By definition, the sum of all the sensitivity indices evaluates to 1, allowing for a straightforward interpretation of each sensitivity index. For instance, if $S_{i}=D_{i}/D=0.75$ , 75% of the model output variance is explained by varying just the parameter $p_{i}$ over its interval. The sum of the first order sensitivity indices also gives useful information regarding the role of pairwise (and higher) interactions of parameters. For example, if the sum of all the first-order sensitivity indices is close to 1, combinations of parameters contribute little to model output variance. Conversely, if the sum of these indices is close to 0, combinations of parameters play a significant role in model output variance.

Open source code exists for implementing variance decomposition methods in MATLAB, including the GSAT package for implementing a Sobol sensitivity analysis (Cannavo, 2012) and eFAST for implementing FAST (Kirschner Lab, 2023). R has a *sensitivity* package that includes an implementation of Sobol sensitivity analysis via the *sobol* command, and an implementation of FAST via the *fast* command (DataCamp, 2023). Independent of the method and language used, care is required in choosing which parameters to analyze and over what range of values (Qian and Mahdi, 2020). As variance-based decomposition methods are computationally expensive, it is not always feasible or necessary to perform a global sensitivity analysis over all dimensions of parameter space. The availability of real-world measurements on parameter values can sometimes remove certain parameters from consideration for a global sensitivity analysis. Alternatively, as recommended by (Zi, 2011), low-cost global sensitivity methods can be used to screen all model parameters and identify those that can be omitted from the more computationally-expensive variance decomposition methods.

Once a subset of parameters has been identified, the lower and upper bound on each parameter must be determined before a Sobol sensitivity analysis can be conducted. The choices made at this step can have a significant impact on your sensitivity analysis (Qian and Mahdi, 2020): choosing a range that is too small can result in underestimating the importance of a parameter on model output variance, whereas choosing a range that is too large to be realistic given the real-world context may result in overestimating the sensitivity index. Unfortunately, there is no correct way to choose these bounds in the absence of good data restricting the value of each parameter, which rarely occurs in more complex biological models. Despite this challenge, understanding the global sensitivity of model parameters, and combinations of model parameters, is essential for generating a heterogeneous set of virtual patients that mimics the variability observed in real-world patients.

Another way to get a more global view of parameter space is through an identifiability analysis, which assesses if the available data results in a model with well-determined parameter values and predictions (Wieland et al., 2021). As noted by Eisenberg and Jain, “Issues of parameter unidentifiability and uncertainty are highly common in mathematical biology—even for very simple models” (Eisenberg and Jain, 2017). The issue tends to be compounded as more complex models are built to capture more mechanistic/physiological detail (Sher et al., 2022). Two types of identifiability are commonly analyzed: structural identifiability and practical identifiability.

Structural identifiability determines if the model structure allows for parameters to be uniquely determined in the context of “perfect” data (Eisenberg and Jain, 2017; Wieland et al., 2021). The exponential growth differential equation in which a population y grows at rate b and dies at rate d :

$$\dot{y}=by-dy$$ (7)

is a trivial example of a model that is not structurally identifiable. Suppose we had error-free, noise-free measurements of the population y that allows one to determine that the net growth rate is 2 (i.e., b−d=2 ). From this perfect data it is not possible to uniquely determine the values of the birth and death rates, and therefore this model is not structurally identifiable. Here, and in general for models that are not structurally identifiable, one can attempt to re-parametrize the system and/or reduce the number of parameters to enforce structural identifiability (Eisenberg and Jain, 2017). In the exponential growth example, simply defining the net growth rate as r=b−d is sufficient to make the model structurally identifiable. Alternatively, one can incorporate the learned growth rate (of 2 in the proposed example) into a formalized relationship between parameters b and d , by replacing d in the model equations with d=b−2 . Either way, the model has been reworked to reduce the number of fitted parameters to achieve identifiability. While structural non-identifiability can be determined *a priori* using only the model, below we will only explore an *a posteriori* method that uses available data. See (Eisenberg and Jain, 2017; Wieland et al., 2021) and the references therein for a summary of *a priori* and *a posteriori* methods for determining structural identifiability.

Practical identifiability determines if the available data, which are invariably incomplete and noisy, are sufficient to determine model parameters with adequate precision (Wieland et al., 2021). As with structural identifiability, there are many approaches for assessing the practical identifiability of model parameters given available data, and reference (Eisenberg and Jain, 2017) gives an overview of several of these methods while providing references to other methods. Herein, we highlight the profile likelihood method, as this approach allows for both the practical and structural identifiability of parameters to be determined (Raue et al., 2009).

The profile likelihood of a parameter $p_{i}$ describes how the goodness of the model fit to data changes as $p_{i}$ is varied across a specified domain. The profile likelihood function for $p_{i}$ can be approximated using the following step-by-step procedure (Raue et al., 2009).

1) Determine the domain over which $p_{i}$ is to be varied. Your local and/or global sensitivity analysis can help here, along with *a priori* biological knowledge.
2) Fix $p_{i}=p_{i}^{*}$ at a value in the domain.
3) Using your chosen optimization algorithm, find the best-fit parameter set, and the associated best-fit value of the cost function, when $p_{i}$ is fixed at $p_{i}^{*}$ .
4) Repeat Steps 2-3 for a discrete set of sampled values $p_{i}^{*}$ adequately covering the pre-determined domain of parameter $p_{i}$ .
5) Plot the optimal value of the cost function determined at Step 3 for each sampled $p_{i}^{*}$ value. The resulting plot is a numerical approximation of the profile likelihood function for parameter $p_{i}$ .

**FIGURE 2:**
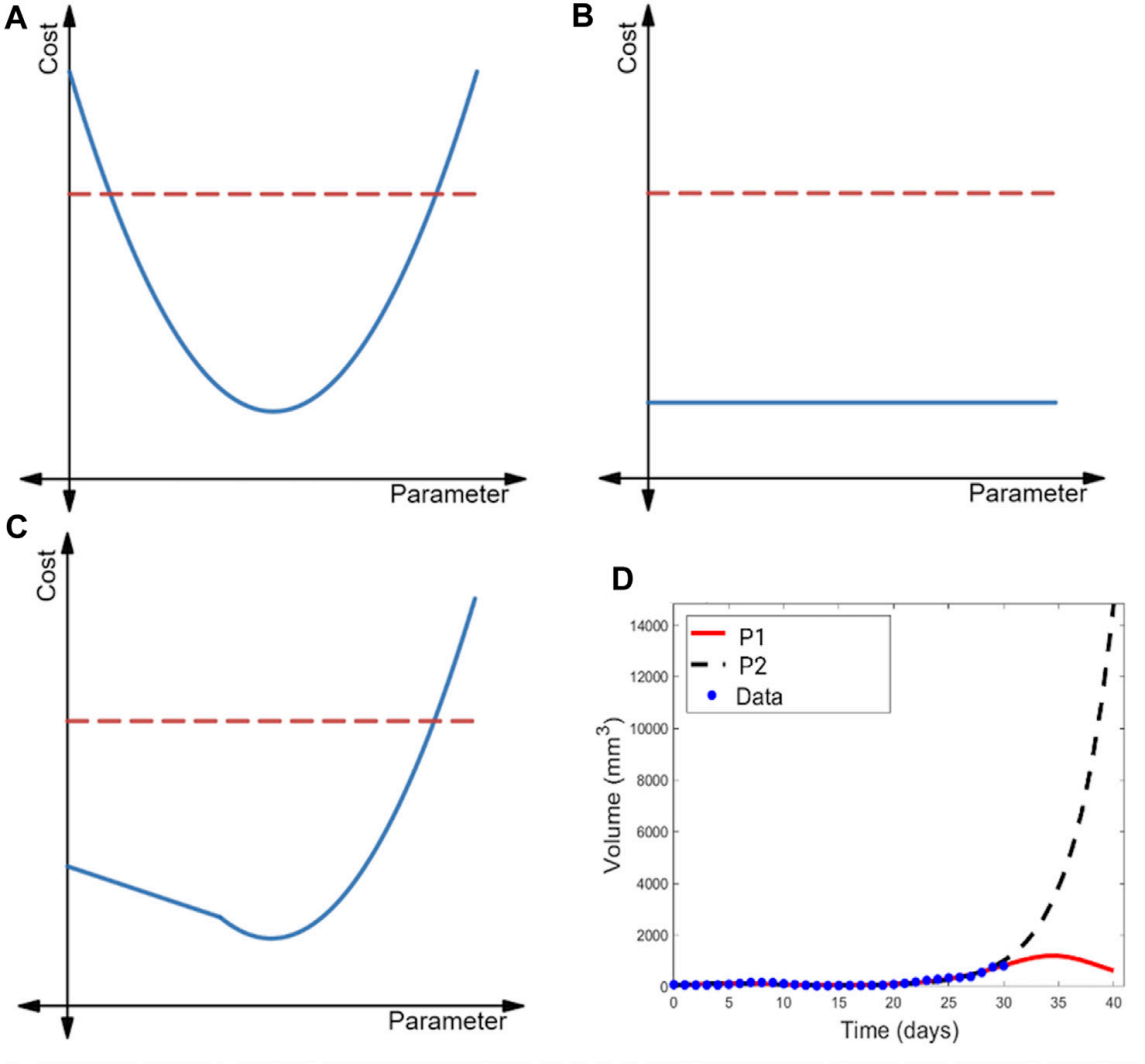
Illustration of the profile likelihood method. Profile likelihood curves (blue) of **(A)** a practically identifiable parameter, **(B)** a structurally non-identifiable parameter, and **(C)** a structurally identifiable but practically non-identifiable parameter. Thresholds for the 95% confidence intervals are indicated with red dashed lines. **(D)** Two parametrizations of the model of immunostimulatory oncolytic viruses and dendritic cell injections in (Luo et al., 2022) that have near-identical fits to tumor volume data, yet yield drastically different predictions for post-treatment tumor dynamics.

A confidence interval for $p_{i}$ can be derived by combining its profile likelihood function with what is called the likelihood threshold. In particular, if k parameters are being fit to profile $p_{i}$ , the 95% likelihood threshold is determined from a chi-squared distribution with significance level α=0.05: $\Delta_{\alpha }=\chi^{2}\left(\alpha ,k\right)$ (Raue et al., 2009). The 95% confidence interval for $p_{i}$ is then the set of all parameters for which the profile likelihood curve falls below the computed threshold $\Delta_{0.05}+\zeta_{\min}$ , where $\zeta_{\min}$ corresponds to the value of the objective function at the best-fit parameter set when fitting all k+1 parameters. Three representative profile likelihood curves and the corresponding 95% confidence threshold are shown in Figures 2A–C.

The profile likelihood curve for a parameter that is practically identifiable given available data should appear near-quadratic (as shown in Figure 2A), with a clear global minimum representing the best-fit value of the parameter. Another feature of a practically identifiable parameter is that its profile likelihood curve exceeds the 95% confidence threshold as the parameter is both decreased and increased from its best-fit value (Raue et al., 2009). In contrast, a structurally non-identifiable parameter is characterized by a completely flat profile likelihood curve (see Figure 2B) (Raue et al., 2009; Eisenberg and Jain, 2017; Wieland et al., 2021), indicating that an infinite set of parametrizations equally well-describe the data. Finally, the profile likelihood of a model parameter that is practically non-identifiable given available data (but is structurally identifiable) does achieve a minimum value. However, as illustrated in Figure 2C, the curve does not exceed the 95% confidence threshold in at least one direction (either when the parameter decreases or increases from its best-fit value) (Raue et al., 2009).

When all parameters in a model are practically identifiable given the available data, one can have high confidence in the value of the model parameters. As a consequence, model outputs tend to be tightly constrained, lending confidence to the model’s predictions (Sher et al., 2022). When a model contains parameters that are practically non-identifiable, a wide range of parametrizations can near equally well-describe the data (Wilkie and Hahnfeldt, 2013; Wilkie and Hahnfeldt, 2017). This may be a benefit when the aim of the analysis is to capture population heterogeneity and to create multiple VPs that will have differing responses in the trial. Caution should be taken, however, as the model may predict an unrealistically wide range of outcomes (Whittaker et al., 2020; Sher et al., 2022), or potentially biologically unfeasible outcomes. In the latter case, the constraints on that parameter could be revisited, or the results taken as an extreme limiting case. In the first case, we may not be able to draw any conclusions about a variable-of-interest in experimental circumstances that differ from the ones in which the data was collected. An example of this is shown in Figure 2D, where a model of combination cancer immunotherapy has multiple parametrizations that well-describe the 31 days of available volumetric time-course data, but extrapolating tumor behavior beyond that time frame results in wildly different predictions of the treatment efficacy and tumor response (Luo et al., 2022).

In summary, sensitivity and identifiability analyses support the work of creating a heterogeneous, yet realistic, group of virtual patients. They do so by helping to refine a fit-to-purpose model that threads the needle between simplicity (more likely with identifiable parameters) and complexity (more detailed yet likely with non-identifiable parameters) (Sher et al., 2022). While models with practically non-identifiable parameters run the risk of predicting unrealistic output, they are also more likely to be able to capture the natural variability observed in real patients (Whittaker et al., 2020). So, as argued by Sher et al. (Sher et al., 2022), one should not dismiss a model with non-identifiable parameters outright, as it may be necessary for representing a heterogeneous (yet realistic) range of behaviors in virtual patients.

### 2.4 Step 4: Generate and verify the virtual patients/parameter sets

Once a model’s parameters have been estimated, and the specific characteristics have been identified to define virtual patients using sensitivity/identifiability analyses, we need to generate a virtual population cohort. In principle, a VP requires *a priori* knowledge of parameter distributions from experimental or clinical data. In practice, however, these data are rarely available, as models frequently include kinetic rate parameters that are difficult or impossible to measure and whose distributions are therefore unknown. To get around this limitation, parameter estimation and sampling are used to produce plausible parameter distributions from which the virtual patients are constructed.

There are three main classes of techniques that can be used: sampling from pre-established distributions (particularly relevant to population pharmacokinetic models), probabilistic interference using, e.g., MCMC, and trajectory matching via global optimization strategies like simulated annealing, genetic algorithms, etc. The choice of VP generation strategy depends on the available data, a modeler’s familiarity with probabilistic inference or optimization approaches, and the goal of the study.

In the most straightforward case, a population pharmacokinetic (PopPK) model has been previously determined from patient data. If one is interested in the effects of PK variability on projected outcomes, a nonlinear mixed effects PopPK model (with any reported correlations between parameters) can be directly implemented (Teutonico et al., 2015; Välitalo et al., 2017; Surendran et al., 2022). From this model, virtual patients can be defined as samples from the previously established log-normal parameter distributions within the PopPK model using multivariate random number generation. However, it is important to note that in most cases, unless coupled with a population pharmacodynamic (PopPD) model, this approach will not provide any information on the effects of physiological variation on PK outcomes.

Unlike sampling from PopPK models that are generally constructed from clinical trial data, MCMC sampling, or Bayesian inference, is a powerful tool that can be used to randomly sample from and reconstruct distributions of parameters. MCMC (and approximate Bayesian computing, as discussed above) is therefore particularly well-suited to generating virtual patients when we have experimental or clinical data for the mechanistic (i.e., non-pharmacokinetic) parameters included in the virtual patient parameter set. Note that approximate Bayesian computing and MCMC have conceptual overlaps, in that they rely on priors for their sampling and use rejection criteria to determine the suitability of proposed posteriors. MCMC uses its sample to compute Bayes’ rule, which is not required in ABC.

MCMC is focused on finding a Markov chain, defined on a state space $s=\left(s_{1},\ldots ,s_{n}\right)$ , that has a stationary distribution that satisfies the probability density function (PDF) of the state space s . The approach makes use of the memoryless nature of Markov chains, their stationary distributions, and the transition probabilities R that describe the probability of moving from state i to state j in the Markov chain. The elements of R are generally unknown but can be constructed using various sampling algorithms. Using such an algorithm (e.g., the Metropolis-Hastings sampling), and sampling from a prior PDF (e.g., a uniform distribution), we can construct a Markov chain that converges to the stationary distribution described above (Brooks et al., 2011). A general sketch of MCMC sampling is as follows:

Suppose we are initially at state $s_{i}$ .

1) Propose a move/jump to new state $s_{j}$ whose conditional probability density given $s_{i}$ is written as $r\left(s_{i},s_{j}\right)$ .
2) Calculate the Hastings ratio (i.e., the probability of acceptance) as

$$a\left(s_{i},s_{j}\right)=\frac{h\left(s_{j}\right)r\left(s_{j},s_{i}\right)}{h\left(s_{i}\right)r\left(s_{i},s_{j}\right)}.$$

Here, $h\left(s_{j}\right)$ is a (unnormalized) density of the stationary distribution of the MCMC sampler.

3) Sample a binomially distributed random variable with success probability $a\left(s_{i},s_{j}\right)$ . If this value is less than $a\left(s_{i},s_{j}\right)$ , the new state is accepted, $s_{i}=s_{j}$ , otherwise keep state $s_{i}$ . The probability $1-a\left(s_{i},s_{j}\right)$ is known as Metropolis rejection.
4) Repeat steps 1-3 until the desired chain length is obtained.

Using this or similar probabilistic inference schemes will provide samples of the model parameters that correspond to the distributions we seek, namely, those in the data of interest.

Unfortunately, parameter distributions are not always known or available from *in vitro*, *in vivo*, or clinical studies. One approach to constructing VPs in this situation is to bootstrap the available experimental data and construct a distribution for each parameter using its best-fit value in each bootstrap replicate, as was done in the Virtual Expansion of Populations for Analyzing Robustness of Therapies method (Barish et al., 2017). A more common approach is to use trajectory matching/parameter fitting optimization to generate parameter samples that enable model outputs to fit the available data. A direct MCMC parameter fitting method will generate parameter sets $p_{i}$ that adequately fit the model to the data and generate the posterior parameter value distributions at the same time. The approach developed by Allen et al. (Allen et al., 2016) and expanded upon by Rieger et al. (Rieger et al., 2018; Rieger et al., 2021), uses global optimization routines (such as MCMC) and selection criteria based on experimental or clinical data to form a VP cohort corresponding to model predictions within a plausible range for the observable outputs. The basic scheme is as follows:

1) Randomly sample selected parameters from uniform (or other) prior distributions centered at their mean values and store in the set $p_{i}$ .
2) For parameter set $p_{i}$ , use a global optimization strategy to minimize the cost function $g\left(p_{i}\right)$ by perturbing $p_{i}$ , where

$$g\left(p_{i}\right)=\underset{j}{\sum }\max\left[{\left(M_{j}\left(p_{i}\right)-\frac{L_{j}+U_{j}}{2}\right)}^{2}-{\left(\frac{U_{j}}{2}-\frac{L_{j}}{2}\right)}^{2},0\right],$$

$p_{i}$ is the resulting fitted parameter vector, $M_{j}\left(p_{i}\right)$ is the model prediction for the $j^{th}$ state variable or output, and $L_{j}$ and $U_{j}$ are biologically plausible lower and upper bounds for the $j^{th}$ model trajectory, respectively. Note that if a trajectory is within the interval defined by the plausible bounds, then $g\left(p_{i}\right)=0$ . Once optimized, store $p_{i}$ as a plausible virtual patient.

3) Repeat steps 1 and 2 until the desired plausible VP pool size is achieved.
4) Select the VP population from the plausible VP pool (i.e., all $p_{i}$ ) to match the prevalence of selected features within the clinical population of interest and adjust the VP population size to correspond to the observed data. This selection step is not always necessary, but can be required when e.g., generating a VP from a larger population than the study population of interest (see Allen et al. (Allen et al., 2016), for example,). Other approaches to pruning candidate VP populations to a final VP are discussed in Derippe et al. (Derippe et al., 2022), where parameter monotonicity is exploited to systematically reduce the relevant parameter space.

In all three scenarios (sampling from previously establish PopPK/PD model, performing MCMC sampling, and using global optimization strategies to match model outputs to clinical observations), it is critical to ensure that the VP population matches observed behaviors. Simulating the entire cohort’s dynamics and validating against secondary data is therefore the final crucial step in the generation procedure (Brady and Enderling, 2019), regardless of the strategy employed.

### 2.5 Step 5: Conduct your virtual clinical trial

Once you have generated your virtual patients, you are ready to explore your problem of interest (Cassidy and Craig, 2019; Jenner et al., 2021a). While there are a large range of questions that one can explore in a virtual trial, here we highlight some examples. Those who work at the interface of preclinical and clinical science may be interested in using a virtual clinical trial to guide dose selection prior to giving the drug to patients. For first in human (FIH) dose selection, the two key criteria that need to be assessed are safety and efficacy. A viable drug candidate will have a minimally efficacious/effective dose that is significantly lower than the maximum tolerated dose; the difference between these two doses is known as the therapeutic index (Tamargo et al., 2015). Safety assessments are typically based on toxicity studies, where increasingly higher doses are given to preclinical species until a safety signal is observed. Pharmacokinetic metrics for these doses, such as the maximum observed concentration ( $C_{\max}$ ), and total exposure (area under the curve, or AUC ) are used to calculate safety margins and to determine dosing thresholds for many drugs.

Minimally efficacious doses are hard to determine since we often do not have clear quantifiable efficacy markers. For this purpose, site of action models have proven to be very helpful. They describe both change in drug concentration over time, and the dynamics of the desired target at the site of action, such as a tumor. With these models, based on known or estimated drug and target properties, one can calculate doses that will result in “sufficient” target coverage. While the level that is sufficient for efficacy may sometimes be hard to quantify precisely, for antagonist drugs, one typically strives to ensure a minimum of 90% target coverage at the site of action. This approach is called a “no regrets” strategy and is predicated on the assumption that if we covered over 90% of the target and have not observed efficacy, then it is most likely the wrong target for this disease, or that targeting this mechanism alone is not sufficient (Kareva et al., 2018; Kareva et al., 2021).

VP-based analysis can allow balancing efficacy and safety considerations in cases when quantifiable safety and efficacy thresholds are available. In such cases, VP simulations can allow estimating at what dose/schedule a majority (often around 90%–95% but it depends on the indication and study design criteria) of simulated patients would be expected to be both as efficacious and safe as possible/acceptable, which may guide dose selection prior to administering the drug to actual patients.

Beyond FIH dose selection, there are many other questions that can be explored in a virtual clinical trial. For instance, virtual clinical trials can be used to identify predictive biomarkers that separate responders from non-responders (Wang et al., 2020; Jenner et al., 2021b; Cardinal et al., 2022). It should be noted, though, that such stratification is only based on inter-individual variability criteria that are already built into the model, and thus VP simulations cannot identify novel factors that may affect treatment safety or efficacy. A virtual clinical trial can also be used to quantify how the optimal personalized treatment protocol varies across VPs (Barish et al., 2017) and to identify promising drug combinations. Further, the generation of VPs can be applied beyond virtual clinical trials to identify biomarkers of disease severity (Jenner et al., 2021a), with the potential to be directly integrated into the clinic and leveraged for drug discovery. The references in this section, along with (Cheng et al., 2022; Surendran et al., 2022), are recommended for the reader who would like to explore concrete examples of executing *in silico* clinical trials.

### 2.6 Step 6: Go back to prior rules as needed and cycle until satisfied

To summarize, designing a virtual clinical trial involves the following steps.

1) Identify the question of interest, such as percent of patients for which a drug is expected to hit an efficacy threshold at a particular dose or schedule.
2) Create a model of sufficient complexity to be able to capture the key features of the process of interest, but not so complex that the variations in output cannot be traced to specific mechanisms built into the model.
3) Parametrize the model using either literature values or experimental data and parameter fitting methods. If the model cannot be parametrized with available data, one may need to return to Step 1 and reframe or simplify the model.
4) Conduct parameter sensitivity and identifiability analyses to help select model parameters that should be held fixed *versus* those used to define the virtual patients.
5) Create a virtual patient cohort.
6) Conduct model simulations and analyses to address the motivating question.
7) If the VP analysis and simulations do not make sense, then a problem has been identified in the process: i) the question was not well posed (in which case return to step 0), ii) the model structure was incorrect (in which case return to step 1), or iii) parameter estimates were incorrect (in which case return to step 2). All of these scenarios can be informative, with the second scenario being perhaps the most scientifically informative, as it suggests that the foundational biological understanding is insufficient. This in turn provides an opportunity to formulate and test additional biological hypotheses using computational methods prior to conducting the VP analysis.

These steps, and the interdependence between them, are summarized in the schematic shown in Figure 1.

Importantly, one should try to find a “test case” scenario to evaluate and calibrate the VP analysis methods. For example, if the virtual population is used to test the impact of a novel checkpoint inhibitor, it would be very informative to check first if the model can capture the key results available for an existing trial of another checkpoint inhibitor, potentially with a similar mechanism of action. If such data are not available, one should still design “control” experiments to evaluate whether the model captures reasonable qualitative behaviors under reasonable “experimental” conditions.

## 3 An illustrative example

**FIGURE 3:**
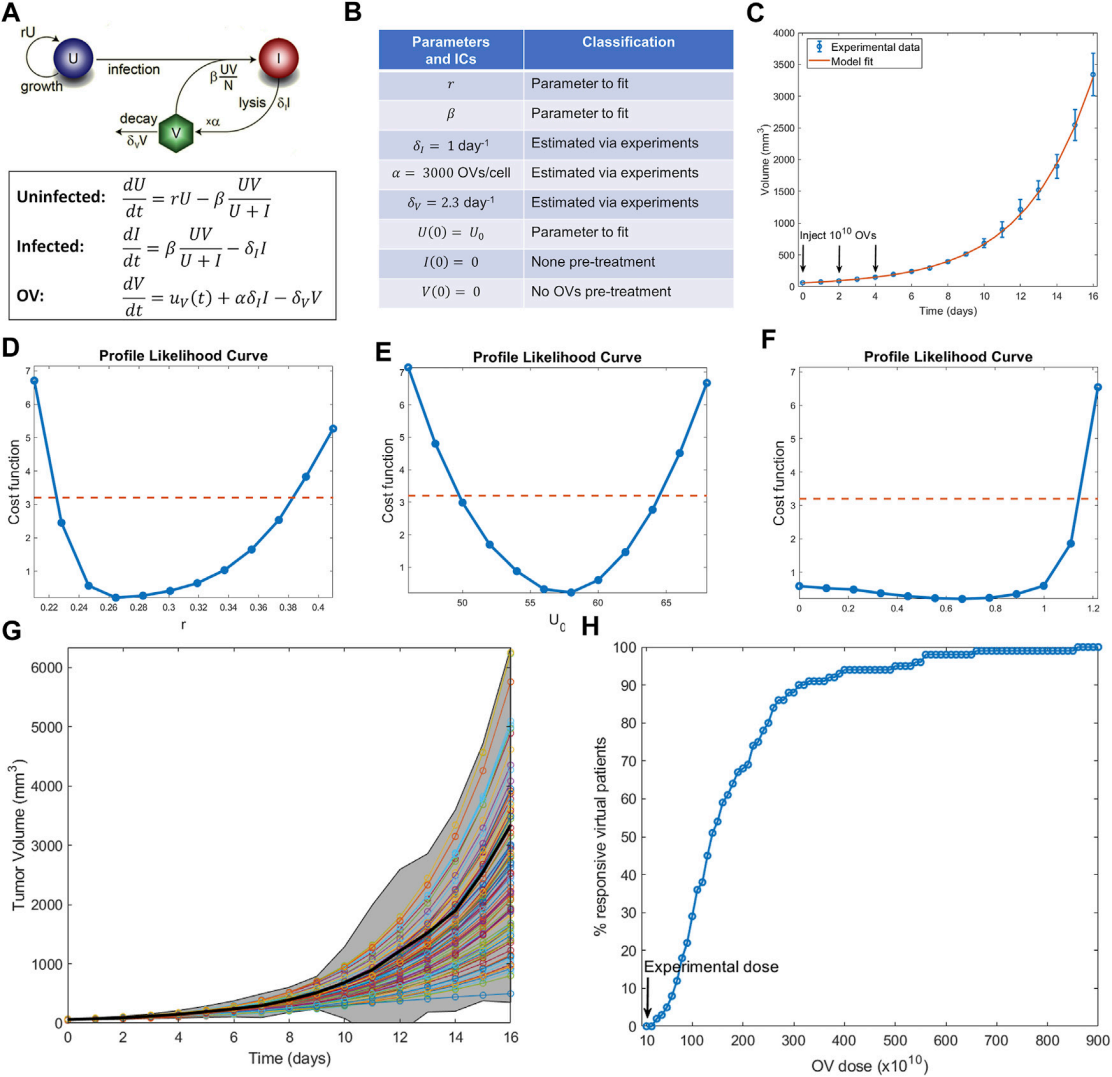
Example of conducting a virtual clinical trial. **(A)** Model of oncolytic virotherapy, where $u_{V}\left(t\right)$ is the source term of the virus determined by the administration schedule shown in **(C)** (Wares et al., 2015). **(B)** Model parameters and initial conditions. **(C)** Best fit of model to average experimental data when fitting only the parameters and initial conditions specified in **(B)**. **(D**–**F)** Profile likelihood curves for the non-fixed parameters. **(G)** 100 virtual patients that form our virtual population. **(H)** Virtual clinical trial predicts what percent of virtual patients shrink in response to OV treatment as a function of OV dose [with the protocol fixed as in (C)]. Doses range from experimental dose of 10^10^ OVs/injection to 90 times the experimental dose.

In this section, we show how to execute the steps described above to conduct a virtual clinical trial using a mathematical model for oncolytic virotherapy. Oncolytic viruses (OVs) are standard viruses that are genetically modified to target, replicate within, and lyse cancer cells (Jenner et al., 2021b). The data used to calibrate the model considers treatment of murine B16-F10 melanoma tumors with three doses of 10^10^ OVs given 2 days apart (Huang et al., 2010). Figure 3A–C shows a previously developed and parametrized three-compartment system of ordinary differential equations to model the OV data (Wares et al., 2015). Parameters that could be estimated from the literature are fixed, whereas parameters that are harder to measure or are likely to vary from patient to patient are fit.

An identifiability analysis of the fit parameters using profile likelihood (Figure 3D–F) demonstrates that the tumor growth rate r and the initial tumor volume $U_{0}$ are practically identifiable, whereas the infectivity parameter β is identifiable structurally but not practically. We were comfortable proceeding with our virtual clinical trial under these conditions, as all parameters are structurally identifiable, and the lack of practical identifiability can result in clinically meaningful response differences between virtual patients. We generated 100 virtual patients by randomly sampling a value of $\left(r,U_{0},\beta \right)$ from a normal distribution with a mean equal to the best-fit value of the parameter ( $\left(\bar{r},\bar{U_{0}},\bar{\beta }\right)=\left(0.2676,0.5693,57.25\right)$ ) and standard deviation equal to a quarter of the mean. Any parametrization for which the corresponding tumor trajectory lies within three standard deviations of the mean trajectory of the experimental data (grey shaded region in Figure 3G) is accepted as a virtual patient. Any trajectories outside the grey region are rejected and not considered for further analysis.

With the virtual population defined, we are ready to conduct a virtual clinical trial. We explored the following question: how does the percent of responders (defined here as individuals whose tumors shrink from it is initial volume) change as a function of the OV dose administered? (Notably, while partial response is defined as at least a 30% decrease in the sum of target lesions according to RECIST criteria (Villaruz and Socinski, 2013), here we define response as any reduction of tumor volume from initial volume, an assumption that can be modified as needed for a particular situation). The results are summarized in Figure 3H. Our virtual clinical trial reveals that OVs must be administered at significantly higher doses than the experimental dose to observe efficacy at the protocol used in (Huang et al., 2010). For instance, giving ten times more OVs per dose only results in 29% of the virtual patients being classified as responders, while a dose 87 times higher than the experimentally tested one is required for all virtual patients to respond. This analysis also suggests that the doses required to elicit a response in a sufficient number of virtual patients are likely prohibitively high. Therefore, either the proposed protocol (three doses given every 2 days) is not appropriate or OVs should not be used as monotherapy. Both possible conclusions provide testable hypotheses that can be evaluated prior to investment into further clinical studies.

## 4 Conclusion

For the last several decades, mathematical modeling has played a pivotal role in the drug development process. It is now poised to further support this process using the novel techniques of *in silico* clinical trials and digital twins. In this review, we focused on virtual clinical trials that aim to simulate and predict the heterogeneity in response across a patient (sub)population. Such *in silico* clinical trials may allow for the drug development process to be more financially efficient, safer, and effective for a broader range of patients. On the other hand, the purpose of a digital twin is to mirror key characteristics of a single person as they pertain to their response to particular therapeutics (22). Together, these computational tools offer immense promise in supporting the development of drugs, doses, protocols, and combinations that benefit larger portions of the patient population.

## Data Availability

The original contributions presented in the study are included in the article, further inquiries can be directed to the corresponding authors.
